# A life-and-death struggle: interaction of insects with entomopathogenic fungi across various infection stages

**DOI:** 10.3389/fimmu.2023.1329843

**Published:** 2024-01-08

**Authors:** Meiqi Ma, Jing Luo, Chong Li, Ioannis Eleftherianos, Wei Zhang, Letian Xu

**Affiliations:** ^1^ State Key Laboratory of Biocatalysis and Enzyme Engineering, School of Life Sciences, Hubei University, Wuhan, China; ^2^ Infection and Innate Immunity Laboratory, Department of Biological Sciences, Institute for Biomedical Sciences, The George Washington University, Washington, DC, United States; ^3^ National Key Laboratory of Green Pesticide, Key Laboratory of Green Pesticide and Agricultural Bioengineering (Ministry of Education), Guizhou University, Guiyang, China

**Keywords:** entomopathogenic fungi, infection stage, insect immunity, defense strategy, pest management

## Abstract

Insects constitute approximately 75% of the world’s recognized fauna, with the majority of species considered as pests. Entomopathogenic fungi (EPF) are parasitic microorganisms capable of efficiently infecting insects, rendering them potent biopesticides. In response to infections, insects have evolved diverse defense mechanisms, prompting EPF to develop a variety of strategies to overcome or circumvent host defenses. While the interaction mechanisms between EPF and insects is well established, recent findings underscore that their interplay is more intricate than previously thought, especially evident across different stages of EPF infection. This review primarily focuses on the interplay between EPF and the insect defense strategies, centered around three infection stages: (1) Early infection stage: involving the pre-contact detection and avoidance behavior of EPF in insects, along with the induction of behavioral responses upon contact with the host cuticle; (2) Penetration and intra-hemolymph growth stage: involving the initiation of intricate cellular and humoral immune functions in insects, while symbiotic microbes can further contribute to host resistance; (3) Host insect’s death stage: involving the ultimate confrontation between pathogens and insects. Infected insects strive to separate themselves from the healthy population, while pathogens rely on the infected insects to spread to new hosts. Also, we discuss a novel pest management strategy underlying the cooperation between EPF infection and disturbing the insect immune system. By enhancing our understanding of the intricate interplay between EPF and the insect, this review provides novel perspectives for EPF-mediated pest management and developing effective fungal insecticides.

## 1 Introduction

Insects constitute the most diverse and abundant clade within the animal kingdom with over a million known species inhabiting our planet, comprising approximately 75% of all described animal species (1, 2). However, nearly half of the known insect species are herbivorous pests as they directly consume crops and indirectly contribute to plant disease transmission (3–6). Currently, the most prevalent method for pest control involves the application of chemical pesticides (7). Nevertheless, concerns have arisen over the pesticide residues, prompting the need for more environmentally friendly and cost-effective pest control strategies (8, 9). Entomopathogenic fungi (EPF) comprise a specialized group of parasitic microorganisms with the remarkable ability to infect and ultimately kill insects and other arthropods. They stand as one of the most potent natural options for pest control, with over 60% of naturally occurring insect diseases attributed to EPF (10–12). These fungi possess several notable characteristics, including environmental adaptation, abundant strain resources, and limited resistance development, which position them as viable alternatives to chemical pesticides in numerous ecosystems (13–16). At present, around 1,000 species of EPF have been identified, and ongoing research continues to unveil new taxa within this group (15, 17, 18). The majority of EPF strains are found in the orders Onygenales, Entomophthorales, Neozygitales, and most prominently, Hypocreales (19, 20). Genera such as *Beauveria*, *Metarhizium*, *Isaria*, *Lecanicilium*, *Nomuraea*, *Hirsutella*, and *Paecilomyces* within the Hypocreales have been extensively studied and some have been developed into commercial EPF agents, particularly within the *Beauveria* and *Metarhizium* genera (21–25).

EPF employ direct penetration of the insect cuticle as their primary mode of infection, although recent studies have indicated that EPF can also utilize oral and respiratory routes to infect their hosts (26, 27). The invasive and developmental processes of EPF can be delineated into six key stages: attachment of conidia to the host, germination, appressorium formation and penetration, fungal growth within the hemolymph, conidia production on host, and ultimately, transmission and dispersal (28, 29). Recent studies have revealed that each step of EPF infection entails the intricate regulation of specific genes, or a combination of various genes, underscoring the existence of highly sophisticated mechanisms employed by EPF to eliminate the targeted insects (28, 30, 31). In the ongoing long-term coevolutionary arms race, insects have developed an array of defense mechanisms to resist potential pathogens. The primary line of defense is the cuticular integument, a robust protective barrier composed of diverse chemical components including n-alkane, fatty acids, chitin, and tanned proteins, which effectively safeguard the internal tissues from the external environment (32, 33). Should EPF breach this barrier, they encounter the second line of defense: the evolutionary conserved innate immune system, which can be categorized into cellular and humoral defenses (34–37).

The complex interaction between EPF and insects signifies a constant battle within the coevolutionary arms race. While previous reviews have detailed the interaction mechanisms, recent findings suggest a greater intricacy, particularly evident across different stages of EPF infection. In this review, we primarily emphasize the interplay between EPF and the insect immune system, focusing on three particular infection stages: (1) Early infection stage: EPF conidia adhere to the host surface and initiate pre-penetration. Insects exploit chemical components, behavioral response, and ectomicrobiomes to defend against EPF infection. (2) Penetration and intra-hemolymph growth phase: EPF penetrate the cuticle, and hyphal bodies grow and produce toxins in the hemolymph. This stage triggers a strong insect cellular and humoral immune response in insects. The involvement of symbiotic microorganisms further complicates the immune interactions. (3) Insect host death phase: this stage marks the ultimate confrontation between EPF and insects. Infected insects strive to isolate themselves from the population to shield it. Meanwhile, EPF secrete antimicrobial products to limit the growth of competitive microbes in the carcass. Finally, we propose a novel pest management strategy that centers around the host-EPF interaction. This involves modifying the EPF through genetic modification in combination with the use of chemicals, dsRNA, or microorganisms to defeat the insect immune system by enhancing EPF virulence.

## 2 During the early infection stage

### 2.1 The adhesion of EPF to the host cuticle and EPF appressorium differentiation

**Figure 1:**
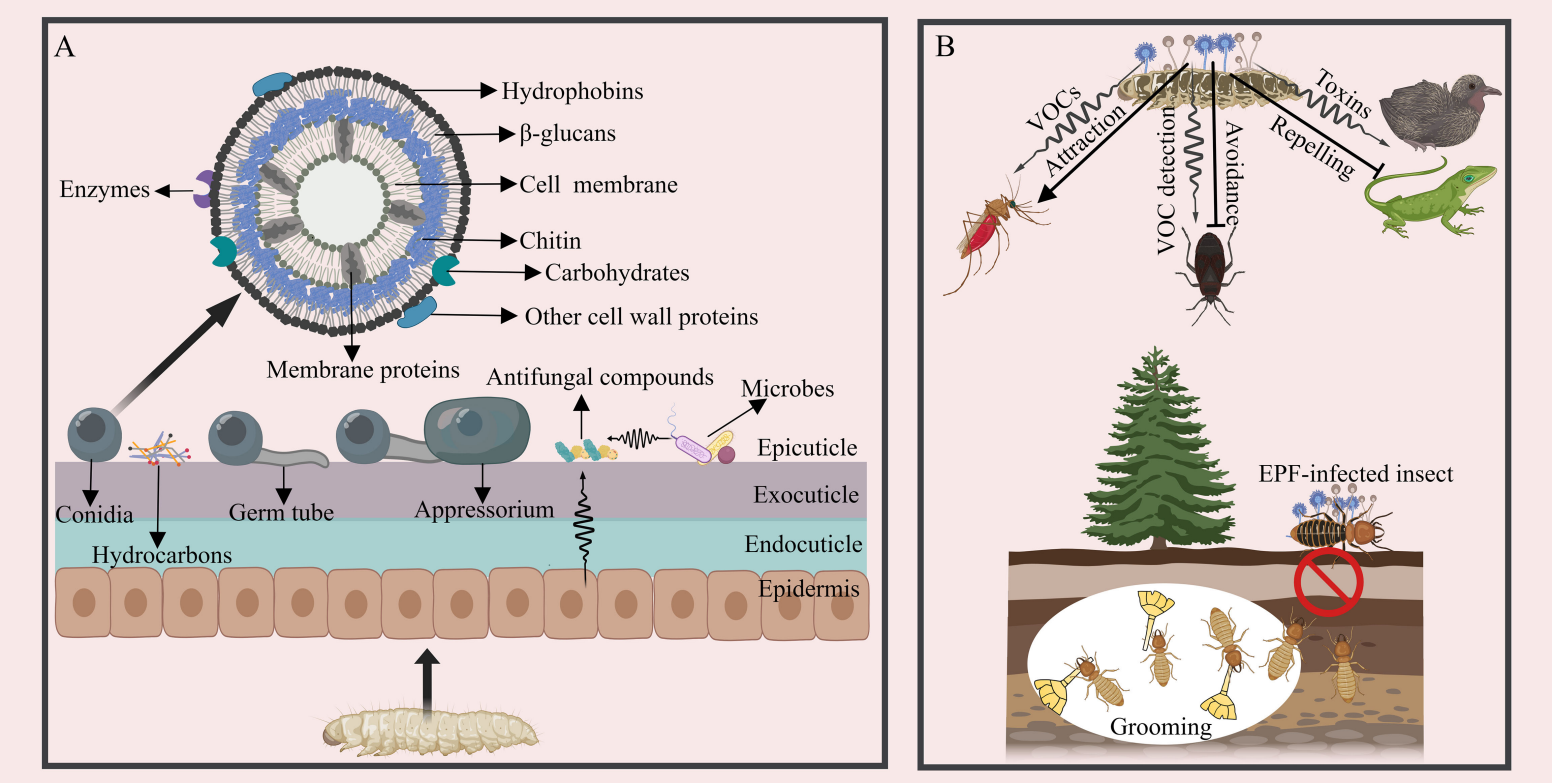
The interaction between EPF and insects during the early infection stage. **(A)** A schematic diagram of fungal adhesion, pre-penetration, and the cuticular defense. The surface of EPF conidia is equipped with hydrophobins and other components facilitating adhesion. The appressorium initiates growth. Antifungal compounds present in the insect cuticle, glandular secretion, and ectomicrobiomes act to inhibit EPF conidial growth. **(B)** Strategies employed by insects to prevent and counteract EPF infection prior to establishment. EPF can attract insects through VOCs, such as acetic acid, 1-octen-3-ol and Chokol K, but insects can detect VOCs from EPF and avoid infection. EPF can also generate toxins to deter other organisms from consuming the infected insect cadavers in the last stage of infection. Social insects exhibit collective behaviors, like grooming and self-removal as a means to circumvent EPF infection.

In the initial stage of EPF infection, the success rate and insect mortality are largely determined by the attachment of single-celled dispersive forms, such as conidia or blastospores, to the host cuticle (38, 39) (**Figure 1A**). Hydrophobins and adhesin proteins on the spores are thought to serve as successive stages in the attachment process: a non-specific (passive) adsorption step followed by a target-specific consolidation phase (40). Different types of fungal propagules exhibit varying adhesive properties: aerial conidia of *B. bassiana* can tightly adhere to hydrophobic surfaces. In contrast, their blastospores quickly bind to hydrophilic surfaces, and submerged conidia adhere to both hydrophobic and hydrophilic surfaces. All the three cell types are infectious, enabling *B. bassiana* to interact with a diverse range of substrates and bind to a broad spectrum of host targets (41, 42). *B. bassiana* possesses two hydrophobins, Hyd1 and Hyd2, localized on the surface of aerial conidia and submerged conidia, but not detected in blastospores. They are responsible for cell surface hydrophobicity, adhesion, and virulence (43, 44). Three adhesin genes (*Adh1-3*) of *B. bassiana* are functionally characterized and *Adh2* plays a role in conidial adherence to insect cuticle (45). In *Metarhizium anisopliae*, two adhesin genes, *Mad1* and *Mad2* have been characterized, and *Mad1* mediates spore adhesion to host surfaces (46). Moreover, several other cell wall proteins are involved in adhesion, for example, the non-hydrophobic cell-wall protein CWP10 of *B. bassiana* enhances conidial adhesion (47). A glycolytic enzyme, glyceraldehyde-3-phosphate dehydrogenase (GAPDH), in *M. anisopliae*, may also contribute to the adhesion of conidia to a host (48). In addition to proteins, the surface of fungal cells contains various carbohydrate moieties, playing diverse roles in adhesion, for example, the negatively charged sugars (sialic acids) on the surface of *Aspergillus fumigatus* are central to conidia adhere to basal lamina proteins (49). Carbohydrates moieties are also the main compounds commonly recognized by host for triggering immune signaling cascades (50, 51). However, carbohydrate analysis is complicated by the complexity of glycan structures and the challenges of separating and detecting carbohydrates experimentally, more investment is needed in this part (52).

Following the attachment of EPF conidia to the host cuticle, appressoria may form at the tips of fungal germ tubes, displaying apical swelling structures adhering to the host cuticle with diverse morphologies (e.g., clavate, spherical, or cup-shaped structures) (53–55) (**Figure 1A**). However, certain EPF species, like *Metarhizium rileyi*, do not undergo appressorium formation, in which the germ tubes grow along the endocuticle and produce lateral branches (56). *M. anisopliae* can occasionally penetrate the cuticle directly without appressoria formation (57). Recent studies have also shown that *B. bassiana* may not always form appressoria during cuticle penetration (55, 58). To date, a series of key genes have been identified in appressoria differentiation like Protein kinase A (PKA) genes in *M. anisopliae* (*MaPKA1*) and *B. bassiana* (*BbMPK1*) (59, 60). In addition, a Rho3 homolog in *Magnaporthe grisea* (*Mgrho3*), G-protein coupled receptors (GPCR) genes and exopolymer galactosaminogalactan (GAG) biosynthetic genes in *Metarhizium* species, as well as the histone lysine methyltransferase *ASH1* in *Metarhizium robertsii* are also essential for appressorium formulation, development and the formation of appressorium mucilage (61–64). Many other genes and pathways involved in regulating fungal sensing and infection of insects are also under investigation (55).

### 2.2 The strategies for insects to avoid

As EPF infect potential hosts, insects have developed strategies to detect and avoid them, both before coming into contact (pre-contact) or after contact (post-contact) (65) (**Figure 1B**). The odorant binding proteins of arthropods can respond actively to volatile organic compounds (VOCs) emitted by EPF, which further alters the insect immune response and induces the repellant effects on the pathogen and modulating host defense (66, 67). These abilities vary among insect species and across different developmental stages. For instance, Japanese beetle (*Popillia japonica*) larvae have been observed to avoid soil containing high concentrations of *M. anisopliae* (68). The generalist predator *Anthocoris nemorum* can detect the presence of *B. bassiana* and actively avoid it (69). Social insects, such as termites and ants, possess the ability to detect EPF and respond with avoidance behaviors. *Reticulitermes flavipes* termites can detect *M. anisopliae*-dusted termites and demonstrate alarm and aggregation reactions (70). The termite *Macrotermes michaelseni* presents similar ability (71, 72); *Coptotermes lacteus* displays an avoidance response by creating short tunnels into substrates containing *M. anisopliae* and sealing them off to prevent further contact with the fungus (73). It is intriguing that EPF can also release VOCs with attractant properties. For instance, female *Anopheles stephensi* mosquitoes are drawn to spores of *B. bassiana* and *M. anisopliae*, as well as other fungal-infected insects, however, the exact mechanisms involved in this behavior remain unclear (74); and the VOCs (such as acetic acid) of the genus *Lecanicillium* can attract female western flower thrips *Frankliniella occidentalis* (75), other VOCs from fungi such as 1-octen-3-ol and Chokol K are also insect attractive signals (76, 77). Additionally, certain plant root-associated EPF can alter host plants traits such as leaf reflectance, attracting herbivorous insects and promoting the EPF dispersal (78). Many phytopathogenic fungi-induced VOCs from plants that can attract insects have been identified (such as methyl salicylate, hexanal and 1-exanol) (79), but whether endophytic EPF can induce plants to produce insect attractive VOCs is less studied.

Moreover, grooming behavior serves as an efficient mechanism for removing conidia after the insect gets in contact with EPF. For example, over 90% of the *M. anisopliae* conidia on the body surface of *Reticulitermes speratus* can be groomed off by their nestmates (80). Mutual grooming behavior is highly effective in protecting *Coptotermes formosanus* termites from *M. anisopliae* infection (81). Similarly, when *Solenopsis invicta* and *Lasius japonicus* are exposed to fungus, both ant species can benefit through exhibiting grooming behavior (82, 83).

### 2.3 In defense against EPF pre-penetration

Upon adherence of EPF, the host cuticle employs various mechanisms to prevent spore adhesion and germination (**Figure 1A**). The inherent hydrophobicity of the insect cuticle generally makes it an ideal substrate for fungal spore adhesion. However, certain insects have developed mechanisms to counteract this trait in order to defend against the adhesion of EPF. For instance, the cuticular fatty amides found in booklice (*Liposcelis bostrychophila*) are able to deter the adhesion of dry EPF conidia by decreasing hydrophobicity and static charge (84). In addition to acting as a physical barrier, the insect cuticle plays a vital role in the molting process. Simple molting can serve as a means to evade infection. For example, rapid ecdysis observed in aphids and the diamondback moth (*Plutella xylostella*) contributes to limiting the EPF ability to infect the host (85, 86). Nevertheless, certain EPF have been observed to hinder the molting process of their hosts by means of oxidative inactivation of host ecdysteroid (87). The hydrocarbons found on the insect cuticle can serve as a carbon source for EPF adhesion and germination, including fatty acids and long chain hydrocarbons. Insects have the ability to alter the hydrocarbon content to evade EPF infection (88). For example, *Metarhizium* is unable to firmly attach to *Aedes aegypti* due to the absence of long-chain hydrocarbons in mosquito larvae, which are probably necessary for fungal development on the host cuticle (89). However, some insect cuticle components exhibit antimicrobial properties, including fatty acids, alkaloids, aldehydes, melanin and antimicrobial peptides (40, 90, 91). Additionally, certain insects release substances such as formic acid, quinones, terpenes, and glucanases, as well as proteinase and chitinase inhibitors, from their epidermal, salivary or poison glands, and these secretions serve to hinder spore adhesion, germination, or appressorium formation (92, 93). For instance, the invasive garden ants, *Lasius neglectus*, produce formic acid as a poison to cleanse the brood and body surfaces, inhibiting the growth of *Metarhizium brunneum* (94, 95). *Tribolium castaneum* produces cuticular secretions containing benzoquinone to inhibit the germination and growth of *B. bassiana* (96). Bedbugs (*Cimex lectularius*) utilize glandular aldehyde secretions, specifically (E)-2-hexenal and (E)-2-octenal, to coat their cuticle and inhibit the growth of *M. anisopliae* (97).

Besides, the ectomicrobiomes, which are resident antagonistic microbes residing on the surface of insects, confer colonization resistance to hosts by producing antimicrobial compounds to inhibit the germination and growth of fungal conidia (40, 58). Surfaces of *Drosophila*, for instance, were found to be densely populated with bacterial cells, which can deter fungal spore germination, and the controlled addition of isolated bacteria in a gnotobiotic setting significantly delays fungal infection of axenic flies (98). Out of 155 bacterial isolates from the cuticle of *Dalbulus maidis* and *Delphacodes kuscheli*, 91 were found to inhibit the growth of *B. bassiana* (99). Antagonistic interactions against fungi have also been observed in ectomicrobiomes residing on leaf-cutting ants (*Acromyrmex subterraneus*) (100), honeybees (*Apis mellifera*) (101), and the oriental fruit moth (*Grapholita molesta*) (102). Intriguingly, the beetle *Lagria villosa* possesses cuticular organs filled with bacterial symbionts, which protect larvae from EPF infection through the production of antifungal compounds (103). Several ant species have the ability to cultivate actinobacteria, which produce potent antimicrobial compounds, in order to safeguard conspecifics and brood from EPF pathogens (104, 105). However, recent research indicates that EPF have evolved strategies to defend against pathogens, for example, *B. bassiana* secrets the defensin-like peptide BbAMP1, which coats fungal spores in order to target and damage Gram-positive bacterial cells, thus suppressing the defensive microbiomes on the host surface (106). This highlights the dynamic and complex interactions between EPF and insect host defenses. Elucidating the critical chemical components, proteins, or ectomicrobiomes that play inhibitory or facilitating roles in EPF infection is essential for the development of highly effective EPF or synergistic agents.

## 3 Fungal penetration and growth in hemolymph and insect immune responses

### 3.1 EPF penetration of the insect cuticle and growth in hemolymph

**Figure 2:**
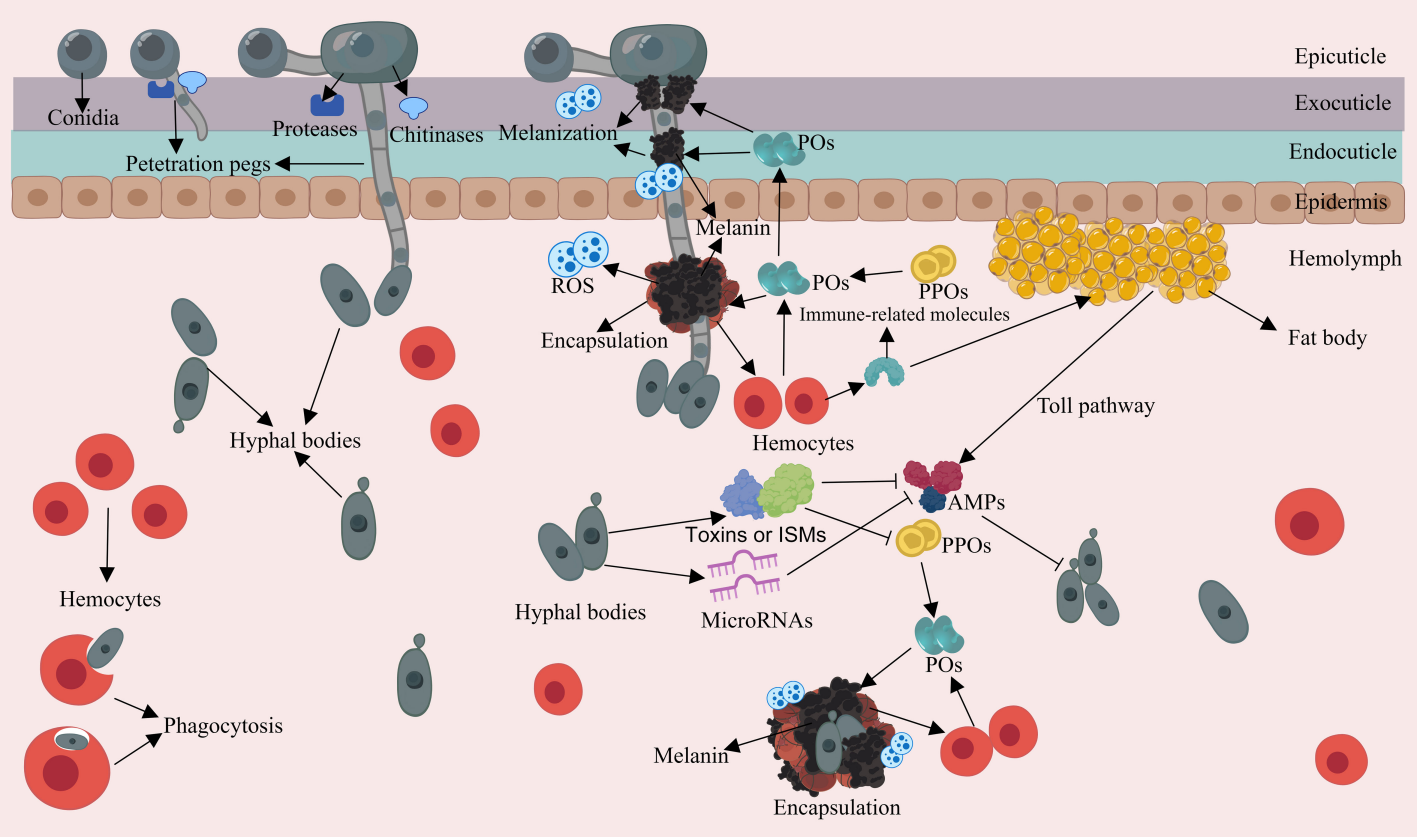
Schematic representation of the interaction between EPF and the insect immune system during the penetration and intra-hemolymph growth phase. Appressoria achieve turgidity and generate penetration pegs. EPF deploy extracellular hydrolytic enzymes to breach the cuticle. Hyphal bodies can produce insecticidal and immunosuppressive metabolites (ISMs), which can deactivate or degrade PPOs, and suppress AMP gene expression. Hyphal bodies can initiate cellular and humoral responses as well as activating the PPO cascade in the compromised insect.

The appressorium is responsible for generating a narrow penetration peg. This peg, under the pressure exerted by the turgor within the appressorium, can generate a downward force to breach the cuticle. Turgor is generated by the accumulation of solutes, cell compartmentalization and water uptake (107–109). Occasionally, the cuticle is slightly distorted at the penetration site, indicating substantial pressure during penetration (57). The penetration of the cuticle is not only achieved through mechanical pressure; it is also facilitated by enzymatic degradation of EPF. The cuticle mainly consists of a chitin framework, tanned proteins and lipids, and EPF are capable of producing a variety of extracellular hydrolytic enzymes that cooperate to degrade the cuticle (110, 111). The most commonly studied proteolytic enzymes are the subtilisin-like serine-protease Pr1 and trypsin-like protease Pr2 in *B. bassiana* and *M. anisopliae* (112). Chitinase genes *chit1*, *chiti2*, and *chit3* in *M. anisopliae* and *Bbchit1* in *B. bassiana* have also been well studied (113–115). It has been demonstrated that overexpressing the Pr‐like protease and chitinase in engineered *B. bassiana* strains significantly accelerates insect death (116). Moreover, EPF have evolved expansions of serine proteases and chitinases to facilitate cuticle degradation (117), and applied research onthese enzymes has been conducted (118). After successfully penetrating the cuticle, hyphae can invade the insect hemolymph (**Figure 2**). Within this environment, EPF generate hyphal bodies, using available nutrients for growth and reproduction through the budding process (119). These hyphal bodies exhibit a distinctive brush-like outermost structure and are believed to have modified carbohydrate epitopes, e.g. lower glucan contents compared to other propagules like submerged conidia and hyphae, which may contribute to their ability to evade host immune responses (31, 50, 120, 121). For example, hyphal bodies of *M. rileyi* induce weaker cellular immune responses in the body cavity of *Helicoverpa armigera* caterpillars compared to *M. rileyi* conidia (122). Additionally, EPF express a range of proteins to coat hyphal bodies, camouflaging their cell wall structures to further evade the host insect immunity (123, 124). An example of such proteins includes LysM domain-containing proteins in *B. bassiana* that bind to chitin in the cell wall to aid in evading insect immunity (125).

### 3.2 Production of insecticidal and immunosuppressive metabolites by EPF

The primary source of nutrition for EPF growth is derived from the insect hemolymph, and the most abundant carbohydrate is the disaccharide trehalose. To access this vital resource, EPF secrete trehalase to break down trehalose and absorb the resulting glucose, or alternatively, they may transport trehalose into their cells for intracellular processing (126, 127). Furthermore, EPF are capable of secreting a range of insecticidal and immunosuppressive metabolites, which serve to hasten the death of the host or interfere with insect immune responses (128) (**Figure 2**). For example, *B. bassiana* produces certain compounds like beauvericin, beauveriolides, 2-pyridone tenellin, and benzoquinone oosporein, which possess insecticidal and cytotoxic properties, expediting fungal colonization (129–132). Cyclopeptide destruxins secreted by EPF such as *M. anisopliae* have been found to deactivate prophenoloxidases (PPOs) and suppress antimicrobial peptide (AMP) gene expression by targeting immunophilins (133, 134). The M35 family of metalloproteases in *M. robertsii* can degrade the PPOs of the host, and *M. robertsii* can directly degrade the inductive antifungal AMPs and protease inhibitors present in insects (135, 136). The extracellular laccase encoded by *BbLac2* in the hyphal bodies of *B. bassiana* can oxidize and deactivate PPOs, as well as eliminate the reactive oxygen species (ROS) in the insect hemocoel (137). Furthermore, microRNA-like RNA is even utilized by EPF to silence the expression of the immune genes of insects, for example, *B. bassiana* exports a microRNA-like RNA (bba-milR1) that can silence expression of the mosquito Toll receptor ligand Spätzle 4 (Spz4), which finally attenuated mosquito immune responses (138). In summary, EPF appear to have evolved effector mechanisms akin to those found in phytopathogens, which serve to disrupt host immunity. Further research in this area is warranted to gain a deeper understanding of these interactions.

### 3.3 Insect immune responses during EPF infection

The insect cuticle serves crucial functions in wound healing and it can also produce immune responses (139). During the EPF penetration, the cuticle triggers a hemostatic response to minimize hemolymph leakage, while simultaneously initiating immune reactions, such as melanization (140) (**Figure 2**). Cuticular melanization is initiated by the PPO activation cascade and involves the conversion of phenolic substrates into black-brown pigments by the phenoloxidase (PO) enzyme (141, 142). In this reaction, ROS and toxic intermediates produced during melanin synthesis can encircle and neutralize pathogens, forming nodules through hemocyte aggregation (143, 144). Furthermore, end-product melanin pigments are important structural and protective elements of the cuticle. The accumulation of melanin in the insect cuticle aids in wound healing and acts as a barrier and toxin to retard cuticular penetration (145, 146). Moreover, hemocytes may migrate to the penetration sites to assist in wound repair and release AMPs by penetrating the basement membrane (33, 147).

When EPF successfully penetrate the cuticle and enter the hemocoel, the insect innate immune springs into action. Through a combination of humoral and cellular responses, along with the PPO cascade, the different layers of the immune system work together to combat the infection (**Figure 2**). This coordinated effort is initiated by the recognition of microbe-associated molecular patterns (MAMPs) by pathogen recognition receptors (PRRs) present in the hemocoel (148–150). As described before, the PPO the cascade is initiated through the interaction of MAMPs with hemocyte-bound PRRs in the hemocoel. Then PO converts phenolic substrates, facilitating the biosynthesis of microbicidal pigment and melanin with the cooperation of hemocytes (142, 151). Mutants with lower PPO activity in *Drosophila* and mosquitoes experience higher mortality when exposed to *Metarhizium* and *Beauveria* pathogens, underscoring the importance of melanization in defending EPF (152, 153).

Humoral immunity in insects engages the fat body in the biosynthesis of antimicrobial effectors such as AMPs and lysozyme (148, 154). These effectors are produced through the activation of Toll and Immune Deficiency (IMD) signaling pathways and are subsequently secreted into the body cavity to combat the invading microbes. Several antifungal peptides have been characterized, such as termicin from termites (155), drosomycin, metchnikowin, Baramicin A and thanatin from *Drosophila* (156–158), heliomycin from *Heliothis virescens* (159), gallerimycin from *Galleria mellonella* larvae (160), cecropins from *Hyalophora cecropia* (161). Mutations in certain genes of the Toll pathway lead to increased sensitivity to EPF infection (162, 163). Moreover, in *Drosophila*, Toll-dependent AMP responses require the Toll ligand Spätzle, indicating that the cellular immune response interacts with the humoral immunity (164).

Notably, the activation of Toll in the fat body and the overexpression of AMPs can lead to reduced glycogen and triglyceride storage in this tissue, negatively affecting the body growth. This suggests that insect immunity places an energy burden on the host (165, 166). However, EPF have evolved strategies to suppress PPO and AMP activities, as discussed above in 3.2 section. The outcome of pathogen-mediated suppression of the immune system’s excessive activation appears to allow insects to conserve energy for growth and development. However, the premise for this hypothesis is contingent on the host insect’s ability to successfully withstand pathogen invasion and proliferation through the restrained immune response.

### 3.4 The insect microbiome regulates the immune interactions between EPF and host

Microbes inhabit various parts of the insect body, including their cuticular surfaces, digestive tissues or cells (endosymbionts), and tract (gut microbiota). Many studies have reported that gut bacteria are essential for host physiology including growth and development, and the establishment and maintenance of the innate immune system (167–172). Interactions between the insect immune system and their EPF may have an impact on the structure of the microbiome, ultimately influencing whether infections are suppressed or promoted. For example, *B. bassiana* can interact with gut microbiota, resulting in EPF-infected mosquitoes containing gut microbiota dying significantly faster than those lacking microbiota, while also increasing the gut microbiota load (173). Similar results have also been observed in *Dendroctonus valens* (174). Conversely, the microbiota can suppress EPF infection. For example, the endosymbiotic bacterium *Wolbachia* in *Drosophila* confers a nonspecific resistance to insect pathogens including *B. bassiana* (175). *Burkholderia* bacterial species inhabiting the midgut crypts of the Southern chinch bug (*Blissus insularis*) can produce antifungal compounds to resist EPF (176). Gut microbiota in *Delia antiqua* assists in inhibiting *B. bassiana* infection, and the removal of the microbiota diminishes larval resistance to fungal infection (177). These effects are due to various EPF metabolites produced during growth in the hemolymph, which modify the expression of a wide range of host insect immune genes (67, 178, 179). The microbes on the cuticle can also shield the host from EPF infection, which has been discussed above in section 2.3.

Interestingly, as mentioned above, in different insects, the interaction between gut microbiota and EPF can have completely opposite effects. The majority of documented instances suggests that the surface microbiota on insects plays a protective role in shielding insects from EPF infections. This effect is likely due to the direct contact between surface microbiota and EPF, leading to more intense competition for nutrients and space between them. In contrast, gut bacteria and EPF have a greater spatial separation (as most EPF initiate infection from the surface) (54). However, it is important to note that these inferences require further investigation.

## 4 The battle between insects and EPF post infection

When the host’s nutrients are depleted and the insect is on the brink of death, the EPF must emerge from the insect to generate and disseminate its conidia. The fungi swiftly transition back to mycelial growth, initiating cuticular penetration once again. Only the hyphae that have successfully breached the insect cuticle are able to generate dispersal conidia, and the genes employed during the host invasion phase are subsequently repurposed for the process, enabling a swift and resource-efficient transformation (28, 31). An illustrative example is *BbMPK1*, known for its crucial role in virulence, as well as in the adhesion and differentiation of appressoria, and penetration of the insect cuticle (180). When mutant conidia lacking *BbMPK1* are injected into the hemocoel, they can generate growing hyphae, but they are incapable of emerging from the surface of insect cadavers (180). Similar factors involved in both inward and outward cuticular penetration have also been explored in *M. acridum* (*MaPls1* gene) (181). This genetic versatility enables a swift and efficient adaptation to new hosts. Conidia regenerated on the host surface are primarily dispersed by non-biological means, such as water and wind (182). The hydrophobic nature of EPF conidia serve not only for promoting the attachment to the insect cuticle, but also for facilitating dispersion via water (183). Additionally, some *Metarhizium* species can produce and accumulate the mycotoxin swainsonine, which protect the insect cadavers from being consumed by birds or other animals (184) (**Figure 1B**). Moreover, other EPF species can behaviorally manipulate infected hosts to ensure dispersal. This phenomenon is exemplified by the enticement of male houseflies (*Musca domestica*) by the cadavers of female flies that have previously been infected with *Entomophthora mascae* (185). Additionally, insect carcasses serve as a nutritional source for the insect or environmental microbiome, typically, only a few other microbes can proliferate from carcasses, and the fungal spores will be laden within the remains of the insect. This suggests that EPF can suppress the competitive proliferation of other microbes in insects or the environment, thus gaining an edge in assimilating host nutrients. For instance, oosporein released from *B. bassiana* have been suggested to participate in inhibiting the growth of bacteria originating from insect cadavers (131), and *B. bassiana* can also be induced to produce iron-chelating 2-pyridones to outcompete other antagonistic microbes (132). The isolation of other microbes with insecticidal activity from the insect carcasses may facilitate the development of synergistic mixtures.

Conversely, various strategies have evolved for infected insects to prevent the further spread of EPF, particularly among social group-living insects. For example, EPF infected individuals distance themselves from the nest to avoid disease transmission among nestmates. Ant workers of *Myrmica rubra* infected by *M. brunneum* are attracted to light, they display a decrease in their attraction towards nestmates or colony odor, and ultimately they withdraw from the nest, possibly because the EPF impair the olfactory system of the infected ants (186). Carpenter ants (*Camponotus aethiops*) infected by *M. brunneum* reduce social interactions and contact with brood, spending most of their time outside the nest until death (187). Sick honey bee workers (*Apis mellifera*) engage in altruistic self-removal, removing themselves from their colony to prevent disease transmission (188). Clearly, this disease-preventing strategy in social insects is detrimental to the dissemination of EPF, but it remains unclear how EPF responds to the defense strategies of social insects. The reasons behind this lack of understanding are uncertain, whether it is due to our limited information or because EPF have not yet evolved effective strategies remains unclear. Besides, behavioral changes in non-social insects to avoid further EPF infection will also be a subject of future investigation.

## 5 Synergy between EPF infection and disruption of insect defensive strategies

In light of our understanding of EPF infection mechanisms and insect anti-EPF defensive strategies, emerging practical evidence suggests that the EPF-mediated disruption of insect immune responses can amplify EPF virulence, offering a promising perspective for refining pest management tactics (**Table 1**). These synergistic approaches could be broadly categorized into four aspects corresponding to EPF infection: 1) Artificially enhancing EPF attachment or penetration capability through various methods. For example, an oil-in-glycerol formulation enhances the lethal infection of *M. anisopliae* conidia to the Red Palm Weevil (*Rhynchophorus ferrugineus*) by promoting adhesion (189); 2) Suppressing or evading host insect immunity through RNAi or specific chemicals. For example, ingestion of bacteria expressing immune-suppressive double-stranded RNAs (dsRNAs) leads to significantly increased mortality in the leaf beetle *Plagiodera versicolora* following *B. bassiana* infection (207). Similar results have been achieved by modifying either EPF or the insect host plant to successfully deliver dsRNA targeting crucial immune genes of the insect (200). 3) Disrupting the detection abilities of social insects to facilitate EPF dispersal. This can be accomplished by genetically modifying EPF to make the pathogens less recognizable to insects, thus avoiding social immunity. Alternatively, disrupting communication in social insects (e.g., using pesticides or dsRNA can increase EPF dissemination within populations) (208, 209). While applications in this area are currently limited, the hypothesis holds significant potential and warrants further exploration. 4) The development of mixed microbial formulations with synergistic effect alongside EPF, such as (*Bacillus thuringiensis* + *Metarhizium* species) pesticides, can offset the limitations of individual pesticides and enhance the control efficiency of EPF in field applications (210, 213). These combined agents present a more socially acceptable strategy compared to genetically modified strains, as they entail lower safety risks.

**Table 1:** The methods to increase EPF infection by disrupting insect defenses.

Categories	EPF species	Content	References
Enhancing EPF virulence through formulations and genetic engineering	*M. anisopliae*	An oil-in-glycerol formulation enhances the adhesion of *M. anisopliae* conidia	(189)
	*M. brunneum*	A Pickering emulsion leads to a two-fold more increase in adhesion of *M. brunneum* conidia	(190)
	*B. bassiana*	A diatomaceous earth can increase *B. bassiana* conidia attachment	(191)
	*Metharizium* and *Beauveria species*	Biopolymer-based formulations improve fungal spore delivery, persistence, and adherence to target insects	(192)
	*B. bassiana*	Expression of a hybrid protease in *B. bassiana* significantly increased fungal virulence by accelerating cuticular penetration	(193)
	*B. bassiana*	Overexpressing both protease and chitinase in *B. bassiana* increased its virulence by accelerating cuticular penetration	(194)
	*M. acridum*	Overexpression of a trehalase (ATM1) accelerated the growth of *M. acridum* in the hemocoel of locusts and improve virulence	(195)
	*M. acridum*	Transferring an esterase gene (*Mest1*) from the generalist *M. robertsii* to the locust specialist *M. acridum* enabled the latter to expand its host range	(116, 196)
Suppressing or evading host insect immunity through genetically engineering and RNAi	*Isaria fumosorosea*	*I. fumosorosea* with *Toll-like receptor 7* targeted dsRNA is more virulent than wild fungus against white fly (*Bemisia tabaci*)	(197, 198)
	*M. robertsii*	*M. robertsii* expressing ds*pr1A* (cuticle-degrading protease Pr1A) and ds*BjαIT* (the scorpion neurotoxin) exhibited an increased virulence	(199)
	*M. anisopliae*	DsRNA-expressed *M. anisopliae* targeting Apolipophorin-D and *Relish* exhibited higher virulence	(200, 201)
	*M. brunneum*	DsRNA-expressed *M. brunneum* targeting insect metalloprotease inhibitor presented an enhanced virulence	(202)
	*B. bassiana*	The spray of dsRNA targeting insect immune genes and *GNBP1* enhanced the virulence of *B. bassiana* in aphid control	(203, 204)
	*B. bassiana*	*B. bassiana* expressing immunosuppressive microRNAs suppressed insect immunity and increased its virulence	(205)
	*Lecanicillium attenuatum*	*L. attenuatum* expressing dsRNAs targeting insect immune genes including *PPO* showed an enhanced virulence	(206)
	*B. bassiana*	Combining *B. bassiana* with immune suppressive dsRNAs expressing bacteria facilitated the fungal infection	(207)
Disrupting the detection abilities of social insects to facilitate EPF dispersal	*B. bassiana*	Expressing a fire ant neuropeptide in *B. bassiana* increased fungal virulence and disrupted the ant’s removal behavior	(208)
	*M. anisopliae*	Upregulating the expression of locust’s OBPs impairs the insect immune responses and alters avoidance behavior	(66)
	*M. anisopliae*	Silencing *Phosphofructokinase* gene disturbed termite social behaviors and weakened its immunity against fungal infections	(209)
Mixed microbial formulations	*M. robertsii*	The synergistic effect between the EPF *M. robertsii* and the bacterium *Bacillus thuringiensis*	(210, 211)
	*B. bassiana*	A mixture of *B. thuringiensis* and *B. bassiana* blastospores	(212)
	*B. bassiana* and *Metarhizium* species	The combination treatments of *B. bassiana*, *Metarhizium* species, and *B. thuringeinsis*	(213, 214)

## 6 Concluding remarks

Overall, this review primarily delves into the interplay between EPF infection and insect defenses, emphasizing three pivotal infection stages. While our comprehension of the immunological interaction between insects and EPF is extensive, there are still some specific pathways that remain elusive. Moreover, the interaction between host-pathogen transcends mere immunological interplay, which encompasses pre-contact communication and the dispersal of EPF post-infection. Their interaction in these stages exhibits a wider array of forms, often involving more species and even producing important ecological or epizootic consequences. A comprehensive understanding of the intricate interplay not only provides molecular insights into fungus-insect interactions but also holds promise for the development of cost-effective and environmentally-friendly pest management strategies.

## Author contributions

MM: Validation, Visualization, Writing – original draft, Writing – review & editing. JL: Writing – original draft, Writing – review & editing. CL: Writing – original draft. IE: Writing – review & editing. WZ: Conceptualization, Funding acquisition, Writing – original draft, Writing – review & editing. LX: Conceptualization, Funding acquisition, Supervision, Validation, Writing – original draft, Writing – review & editing. IE:.
